# Inducing Antigen‐Specific and Functional Immune Responses in Mice Toward Bovine Herpesvirus 1 and Bovine Respiratory Syncytial Virus by Chimeric Peptides Delivered by Bovine Herpesvirus 4‐Based Vector

**DOI:** 10.1111/imm.70148

**Published:** 2026-05-15

**Authors:** Antonino Di Lorenzo, Sergio Minesso, Chiara Cossu, Valentina Franceschi, Elisabetta Bolli, Vittorio Madia, Laura Conti, Gaetano Donofrio

**Affiliations:** ^1^ Department of Molecular Biotechnology and Health Sciences Molecular Biotechnology Center “Guido Tarone”, University of Turin Torino Italy; ^2^ Department of Veterinary Science University of Parma Parma Italy

## Abstract

Bovine respiratory disease (BRD) remains a major health and economic challenge for the cattle industry, driven by the interplay of viral and bacterial pathogens that compromise animal welfare, productivity and antimicrobial stewardship. Among the primary viral agents, Bovine herpesvirus 1 (BoHV‐1) and Bovine respiratory syncytial virus (BRSV) play critical roles in initiating and exacerbating respiratory pathology. In this study, we engineered two recombinant Bovine herpesvirus 4 (BoHV‐4)‐based vectors encoding chimeric antigens based on BoHV‐1 glycoprotein D (gD) and BRSV fusion glycoprotein (gF), with the goal of developing a multivalent vaccine platform. Immunological evaluation in a murine model demonstrated that both vaccine constructs elicited robust humoral and cell‐mediated immune responses directed against both pathogens. Vaccination induced neutralising antibodies capable of inhibiting BoHV‐1 and BRSV infection, as well as antigen‐specific T‐cell responses that mediated cytotoxic activity against target cells expressing either antigen. These findings provide proof‐of‐concept that chimeric antigens are effective in eliciting humoral and cellular immune response toward two main different pathogens, BoHV‐1 and BRSV, and BoHV‐4 is a versatile vector for the delivery of heterologous antigens. The demonstrated ability to induce both virus neutralisation and cytotoxic T‐cell activity supports the further development of BoHV‐4‐vectored bivalent vaccines for BRD control, with potential application at improving livestock health and reducing reliance on antimicrobial treatments. Moreover, the present study highlights BoHV‐4‐based vectors and chimeric peptides as a promising bivalent vaccine platform potentially translatable for controlling similar viruses like human respiratory syncytial virus (hRSV) and varicella zoster virus (VZV) across human populations.

## Graphical abstract

Bovine Alphaherpesvirus 1 (BoHV‐1) and Bovine respiratory syncytial virus (BRSV) are major drivers of respiratory disease in cattle. (A) To target both pathogens, two chimeric antigens—membrane‐linked and secreted—combining BoHV‐1 glycoprotein D and BRSV fusion glycoprotein were designed and expressed using recombinant BoHV‐4‐based vectors. (B) These constructs induced robust humoral and cellular immunity, including neutralising antibodies and antigen‐specific cytotoxic T cells. This strategy represents a promising one health approach with potential applications for controlling related human viruses such as hRSV and VZV.

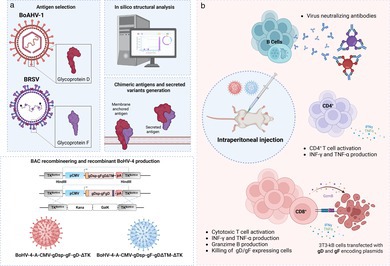

## 1 Introduction

Bovine respiratory disease (BRD) is one of the most significant causes of economic loss in the cattle industry worldwide, contributing to decreased productivity, increased antimicrobial use, and elevated mortality in feedlot and dairy operations. The disease is multifactorial, involving viral and bacterial pathogens as well as stress factors and detrimental environmental conditions. The onset of BRD typically involves a primary viral infection that weakens the host's immune defences, thereby creating a favourable environment for subsequent secondary bacterial infection [1]. Among the primary viral pathogens involved in BRD, Bovine herpesvirus 1 (BoHV‐1), the causative agent of infectious bovine rhinotracheitis (IBR), and Bovine respiratory syncytial virus (BRSV) play critical roles by both initiating and exacerbating respiratory infections, particularly in young calves [1, 2]. BoHV‐1 is a member of *Herpesviridae* family and *Alphaherpesvirinae* subfamily. BoHV‐1 glycoprotein D (gD) is essential for viral envelope fusion with the host cell membrane and has been regarded as a major target in vaccine design due to its ability to stimulate both cell‐mediated and humoral immune responses, including the production of neutralising antibodies [3]. BRSV belongs to the *Pneumoviridae* family, *Orthopneumovirus* genus and *Mononegavirales* order, a group of enveloped negative‐sense RNA (ssRNA−) viruses. BRSV glycoprotein F (gF) has a critical role in both attachment and fusion of viral envelope with the host cell membrane: gF binds to a specific receptor on respiratory epithelial cells triggering a conformational change which leads the fusion peptide of the gF to be inserted in the host cell membrane allowing syncytium formation, the peculiar fusion of infected cells, from which BRSV derives its name [4]. Due to its critical role in initiating infection, high conservation among BRSV isolates, and the presence of multiple neutralising epitopes, BRSV gF has become a strong candidate for vaccine development [5, 6].

In the context of BRD, controlling viral infections in cattle not only improves animal welfare and food production sustainability but also reduces the need for antibiotics, thus limiting the risk of antimicrobial resistance (AMR)—a growing global health concern [7]. Furthermore, several bovine respiratory viruses, including BRSV, share similarities with human pathogens (e.g., hRSV), making them valuable comparative models for vaccine development and immunological studies [8, 9, 10].

Current strategies for BRD prevention rely on multivalent vaccine formulations combining live‐attenuated, inactivated, and recombinant proteins from BRD pathogens, typically including modified live BoHV‐1 and BRSV strains [11]. Despite the availability of commercial vaccines, protection is often incomplete due to limited immunogenicity, short duration of immunity and interference from maternal antibodies [12].

Viral vector vaccines represent an innovative platform in veterinary vaccinology, capable of inducing both humoral and cellular immune responses, while enabling the development of multivalent vaccines and compatibility with DIVA (differentiation of infected and vaccinated animals) strategies through targeted manipulation of antigens [13]. BoHV‐4, a gammaherpesvirus with low pathogenicity and a broad cellular tropism, has emerged as a promising viral vector for the delivery of heterologous antigens in veterinary vaccinology [14, 15, 16]. Its ability to establish persistent infection without causing overt disease, coupled with a large genome suitable for foreign gene insertion, makes it an attractive candidate for the development of multivalent vaccines.

In this study, we report the construction and immunological evaluation of a BoHV‐4‐based bivalent vaccine vector expressing immunogenic antigens derived from BoHV‐1 gD and BRSV gF chimerization. While the goal is to provide cross‐protection in cattle, we first assessed vaccine‐induced immune responses in a murine model, providing a proof‐of‐concept for the platform's immunogenicity. Our findings support the continued development of chimeric peptide‐based approaches in combination with BoHV‐4‐vectored vaccines to enhance the prevention and control of respiratory diseases in livestock populations, while potentially informing strategies against related pathogens of human relevance.

## 2 Results

### 2.1 Topological Prediction and Rational Design of Chimeric Peptides

**FIGURE 1:**
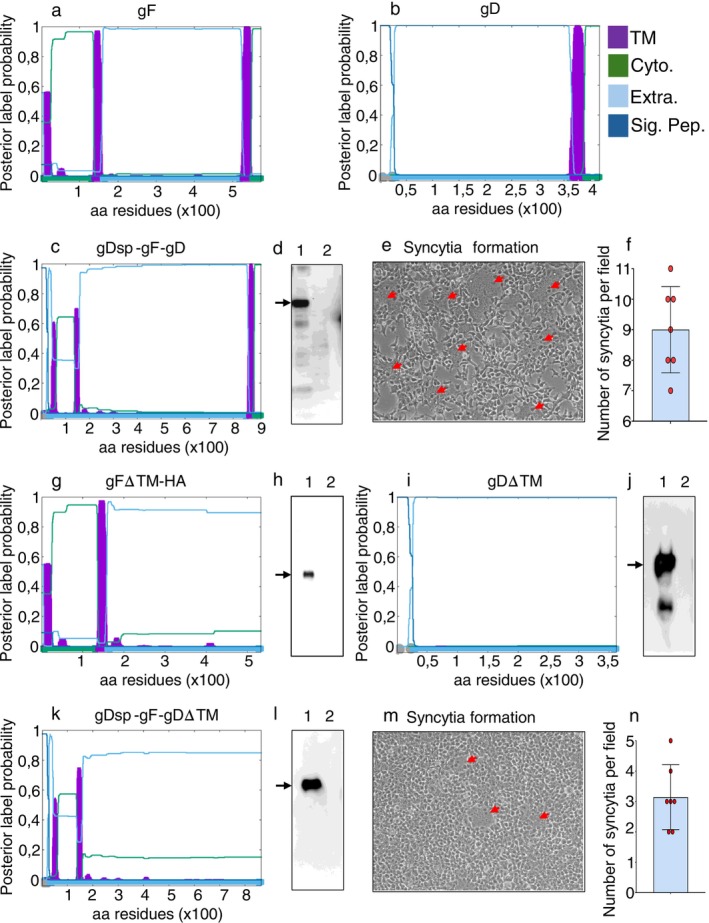
Topological prediction and rational design of chimeric peptides. (a–c) Server output prediction of transmembrane topology and signal peptides from the amino acid sequence of gF, gD and gDsp‐gF‐gD proteins respectively. The plot shows the posterior probabilities of cytoplasmic (Cyto., green)/extracellular (Extra., azure)/transmembrane helix (TM., purple)/signal peptide (Sig. Pep., blue) by calculating the total probability that a residue belongs to a helix, cytoplasmic, or non‐cytoplasmic summed over all possible paths through the model. (d) Western immunoblotting of pINT2‐CMV‐gDsp‐gF‐gD transfected HEK 293T cells extracts (lane 1). Lanes were loaded with 20 μg of total protein cell extract; cells transfected with pEGFPC‐1 served as negative controls (Mock, 2). (e) Representative phase contrast (10×) image of pINT2‐CMV‐gDsp‐gF‐gD transfected HEK 293T cells with syncytia indicated by red arrows and (f) quantified counting the number of syncytia per field (seven fields per well were counted and the experiment was repeated 3 times). (g) Server output prediction of transmembrane topology and signal peptides from the amino acid sequence of gFΔTM‐HA. (h) Western immunoblotting of pINT2‐gFΔTM‐HA transfected HEK 293T cells (lane 1). Lanes were loaded with 20 μL of clarified transfected cells serum free medium supernatant; clarified transfected cells serum free medium supernatant from pEGFPC‐1 transfected cells served as negative controls (Mock, 2). (i) Server output prediction of transmembrane topology and signal peptides from the amino acid sequence of gDΔTM. (j) Western immunoblotting of pINT2‐gDΔTM transfected HEK 293T cells (lane 1). Lanes were loaded with 20 μL of clarified transfected cells serum free medium supernatant; clarified transfected cells serum free medium supernatant from pEGFPC‐1 transfected cells served as negative controls (Mock, 2). (k) Server output prediction of transmembrane topology and signal peptides from the amino acid sequence of gDsp‐gF‐gDΔTM. (l) Western immunoblotting of pINT2‐gDsp‐gF‐gDΔTM transfected HEK 293T cells (lane 1). Lanes were loaded with 20 μL of clarified transfected cells serum free medium supernatant; clarified transfected cells serum free medium supernatant from pEGFPC‐1 transfected cells served as negative controls (Mock, 2). (m) Representative phase contrast (10×) image of pINT2‐CMV‐gDsp‐gF‐gD transfected HEK 293T cells with syncytia indicated by red arrows and quantified (n) counting the number of syncytia per field (seven fields per well were counted and the experiment was repeated 3 times).

According to predictions by Phobius (https://phobius.sbc.su.se/), a free online tool for combined transmembrane topology and signal peptide prediction, and consistent with previously published research [9, 17, 18], both BRSV gF (Figure 1a) and BoHV‐1 gD (Figure 1b) are classified as type‐I transmembrane proteins. BRSV gF features an amino‐terminal signal peptide (aa1 to aa25), an extracellular domain (aa26 to aa524), a hydrophobic region responsible for fusogenic activity (aa137 to aa158), a transmembrane domain (aa525 to aa551) and a cytoplasmic domain (aa552 to aa574). The activation of BRSV gF for membrane fusion necessitates cleavage by furin‐like protease at two sites (R/G: aa109/aa110; R/F: aa136/aa137), which removes p27 and separates the protein into the disulfide‐linked F1 (aa137 to aa524) and F2 (aa26 to aa109) (Figure 1a). BoHV‐1 gD, more simply, possesses an amino‐terminal signal peptide (aa1 to aa25), an extracellular domain (aa26 to aa339) a transmembrane domain (aa365 to aa388) and a cytoplasmic tail (aa339 to aa417) (Figure 1b). Given that the task was to chimerize gF with gD, considering that gF undergoes more complex post‐translational processing than gD (including the formation of F1 and F2 and the elimination of p27), it was decided to position gF at the amino‐terminal relative to gD. The signal peptide prediction score for gF, as determined by Phobius, was lower compared to that of gD (Figure 1a,b). Therefore, the gF signal peptide (aa1 to aa27) was substituted with the gD signal peptide (aa1 to aa27), creating gDsp‐gF. Furthermore, gDsp‐gF, lacking the transmembrane domain and cytoplasmic tail (aa525 to aa574), was fused with gD, which was deprived of its signal peptide (aa26 to aa417). This chimeric protein, referred to as gDsp‐gF‐gD, retained all the topological features of gF and gD, with a significant increase in the signal peptide prediction score (Figure 1c). Before generating a suitable expression cassette for the gDsp‐gF‐gD ORF gene in mammalian cells, the nucleotide composition of the gDsp‐gF‐gD gene was analysed to improve expression in mammalian cells. The gDsp‐gF‐gD codon usage was first adapted to the human genome using the Jcat codon adaptation tool (http://www.jcat.de/). Starting from the human‐adapted gDsp‐gF‐gD ORF, a Kozak sequence (to enhance translation) and two restriction enzyme sites (to facilitate sub‐cloning into a suitable vector) were added at the ends of the ORF. The synthetic gDsp‐gF‐gD ORF was placed under the transcriptional control of the CMV promoter and the SV40 polyadenylation signal to create the pINT2‐CMV‐gDsp‐gF‐gD construct. Transient transfection of HEK 293T cells with pINT2‐CMV‐gDsp‐gF‐gD resulted in the expression of gDsp‐gF‐gD, as confirmed by Western blotting (Figure 1d) and immunofluorescence using a monoclonal antibody that recognises an epitope at the carboxy‐terminal of gD. gDsp‐gF‐gD retained fusogenic activity conferred by gF, as shown by the large number and size of syncytia observed in pINT2‐CMV‐gDsp‐gF‐gD transfected cells (Figure 1e,f), whereas no syncytia were observed in pEGFP‐C1 transfected cells (data not shown).

Given the varied processing and presentation of protein antigens by antigen‐presenting cells (APCs) to the cells of adaptive immunity, which depends on their subcellular localisation and whether they are membrane‐linked or secreted, it was reasoned the potential generation of a secreted form of gDsp‐gF‐gD. Based on the understanding that type‐I transmembrane proteins can be secreted once they lose their transmembrane domain, the secreted forms of gF and gD were individually investigated before attempting to generate gDsp‐gF‐gD as a secreted peptide. Additionally, the secreted forms of gF and gD represent valuable tools for immunological assays during in vitro and in vivo immunological studies. Removing the transmembrane domain and cytoplasmic tail from gF (aa525 to aa574) makes its secretion unpredictable due to hydrophobic regions in the amino terminus (Figure 1g), unlike gD (Figure 1i). When the transmembrane domain and cytoplasmic tail were deleted from gF and it was tagged with HA (gFΔTM‐HA), it was efficiently secreted into the medium of transfected mammalian cells, as confirmed by western immunoblotting using an anti‐HA monoclonal antibody (Figure 1h). A similar result was observed for gD with the transmembrane domain and cytoplasmic tail deleted (gDΔTM) (Figure 1j). In this instance, tagging gDΔTM was unnecessary because a monoclonal antibody (mAb) targeting an epitope in the amino terminus of gD was available. Based on this initial information, a secreted version, gDsp‐gF‐gDΔTM, was created by removing the transmembrane domain from gDsp‐gF‐gD. As predicted (Figure 1k), gDsp‐gF‐gDΔTM was efficiently secreted (Figure 1l) into the medium of transfected mammalian cells, as confirmed by western immunoblotting using an anti‐gD mAb. Syncytia formation activity was maintained, although to a lesser extent compared to gDsp‐gF‐gD (Figure 1m,n) (an average of 3 syncytia per field for gDsp‐gF‐gDΔTM and 9 for gDsp‐gF‐gD; *p* < 0.0001 as measured by *t*‐test with GraphPad Prism). This is likely attributed to the hydrophobic nature of the gD transmembrane domain. However, this aspect was beyond the scope of our study and warrants further investigation.

### 2.2 BoHV‐4‐A‐CMV‐gDsp‐gF‐gDΔTK and BoHV‐4‐A‐CMV‐gDsp‐gF‐gDΔTM‐ΔTK Generation

**FIGURE 2:**
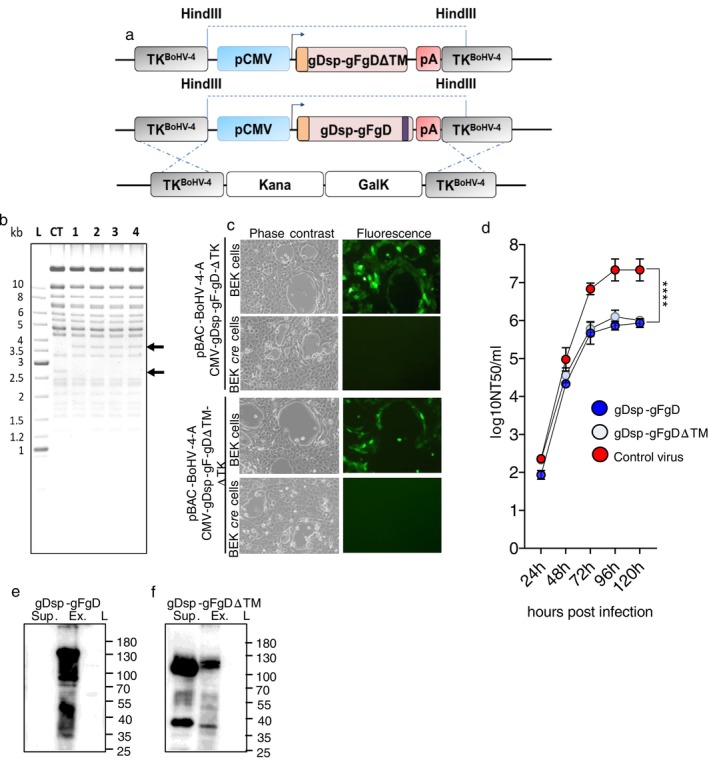
Generation and characterisation of BoHV‐4‐A‐CMV‐gDsp‐gF‐gD‐ΔTK and BoHV‐4‐A‐CMV‐gDsp‐gF‐gDΔTM‐ΔTK. (a) Diagram (not to scale) describing the re‐targeting event obtained by heat‐inducible homologous recombination in SW102 containing pBAC‐BoHV‐4‐A‐TK‐KanaGalK‐TK, where the Kana/GalK cassette was replaced with CMV‐gDsp‐gF‐gD or CMV‐gDsp‐gF‐gDΔTM expression cassettes flanked by BoHV‐4 TK sequences, located in pINT2 shuttle plasmid vector (pINT2‐CMV‐gDsp‐gF‐gD or pINT2‐CMV‐gDsp‐gF‐gDΔTM). (b) Representative 2‐deoxy‐galactose resistant colonies pBAC‐BoHV‐4‐A‐CMV‐gDsp‐gF‐gDΔTM‐ΔTK (1–4) tested by HindIII restriction enzyme analysis, agar gel electrophoresis and compared with the parental pBoHV‐4‐A‐Kana/GalKΔTK (CT (Control)). The 2650 bp band, corresponding to the un‐retargeted pBAC‐BoHV‐4‐A‐TK‐KanaGalK‐TK control (CT; indicated by an arrow) has been replaced by a 3900 bp band in BoHV‐4‐A‐CMV‐gDsp‐gF‐gDΔTM‐ΔTK (1–4 indicated by an arrow). Similarly, for pBAC‐BoHV‐4‐A‐CMV‐gDsp‐gF‐gDΔTK, the 2650 bp band has been replaced by a 4064 bp band (data not shown). (c) Representative images of phase contrast and fluorescent fields of plaques formed by viable reconstituted recombinant BoHV‐4‐A‐CMV‐gDsp‐gF‐gD‐ΔTK and BoHV‐4‐A‐CMV‐gDsp‐gF‐gDΔTM‐ΔTK after the corresponding BAC DNA was electroporated into BEK cells or in BEK cells expressing cre recombinase (Magnification, ×10). (d) Replication kinetics of BoHV‐4‐A‐CMV‐gDsp‐gF‐gD‐ΔTK (blue circle) and BoHV‐4‐A‐CMV‐gDsp‐gF‐gDΔTM‐ΔTK (azure circle) growth on BEK cells and compared with the parental BoHV‐4‐A isolate (red circle). The data presented are the means ± standard errors of triplicate measurements (*p* < 0.0001 as measured by two‐way ANOVA followed by Tukey's test for multiple comparisons). (e) Western immunoblotting of supernatant (Sup.) or extract (Ex.) from cells infected with BoHV‐4‐A‐CMV‐gDsp‐gF‐gD‐ΔTK (hereafter called gDsp‐gFgD) or (f) BoHV‐4‐A‐CMV‐gDsp‐gF‐gDΔTM‐ΔTK (hereafter named gDsp‐gFgDΔTM). The lanes were loaded with 20 μg of protein extract or 20 μL of supernatant. L indicate the Mass Ladder.

When the expression cassettes for gDsp‐gF‐gD and gDsp‐gF‐gDΔTM are transfected into mammalian cells, they are effectively expressed (Figure S1). To assess their immunogenicity in vivo, various delivery platforms such as DNA, mRNA, protein, or viral vectors can be used. The BoHV‐4‐based viral vector platform was chosen due to its specific characteristics, making it suitable for future use as a vaccine in bovines, as gF and gD are immunodominant antigens from pathogenic viruses that infect bovines. BoHV‐4 thymidine kinase (TK) genomic region is highly conserved among BoHV‐4 isolates, ensuring the stability of the genomic locus for inserting foreign expression cassettes. The BoHV‐4 TK gene was disrupted by inserting foreign sequences, which did not interfere with viral replication in vitro. As a result, the BoHV‐4 TK gene was chosen as the target site for inserting the CMV‐gDsp‐gF‐gD and CMV‐gDsp‐gF‐gDΔTM expression cassettes into the BoHV‐4‐A genome, cloned as a BAC. The BAC recombineering system approach, modified with a kanamycin selection step, was employed for this purpose as previously described [19]. Re‐targeting was conducted to replace the KanaGalK selection cassette with pINT2‐CMV‐gDsp‐gF‐gD (TK‐CMV‐gDsp‐gF‐gD‐TK) or pINT2‐CMV‐gDsp‐gF‐gDΔTM (TK‐CMV‐gDsp‐gF‐gDΔTM‐TK) (Figure 2a,b). Negative selection on deoxygalactose (DOG) minimal plates, following a negative selection with medium containing kanamycin, allowed the isolation of positive clones for the correct targeting. Subsequently, the pBAC‐BoHV‐4‐A‐CMV‐gDsp‐gF‐gD‐ΔTK and pBAC‐BoHV‐4‐A‐CMV‐gDsp‐gF‐gDΔTM‐ΔTK were transfected into BEK cells, resulting in viable virus (Figure 2c). Roughly 1.5 log of difference, in terms of growth characteristics, were observed between BoHV‐4‐A‐CMV‐gDsp‐gF‐gD‐ΔTK, BoHV‐4‐A‐CMV‐gDsp‐gF‐gDΔTM‐ΔTK and BoHV‐4‐A (Figure 2d). This observation may be explained by overexpression of the chimeric peptides, leading to a partial toxic effect in infected cells.

Cell lines infected with BoHV‐4‐A‐CMV‐gDsp‐gF‐gD‐ΔTK robustly expressed gDsp‐gF‐gD only in the cell extract and gDsp‐gF‐gDΔTM BoHV‐4‐A‐CMV‐gDsp‐gF‐gDΔTM‐ΔTK in both the cell extract and supernatant, as shown by western immnoblotting (Figure 2e,f).

### 2.3 BoHV‐4‐A‐CMV‐gDsp‐gF‐gD‐ΔTK (gDsp‐gFgD) and BoHV‐4‐A‐CMV‐gDsp‐gF‐gDΔTM‐ΔTK (gDsp‐gFgDΔTM) Produce Effective Immunization in Mice

**FIGURE 3:**
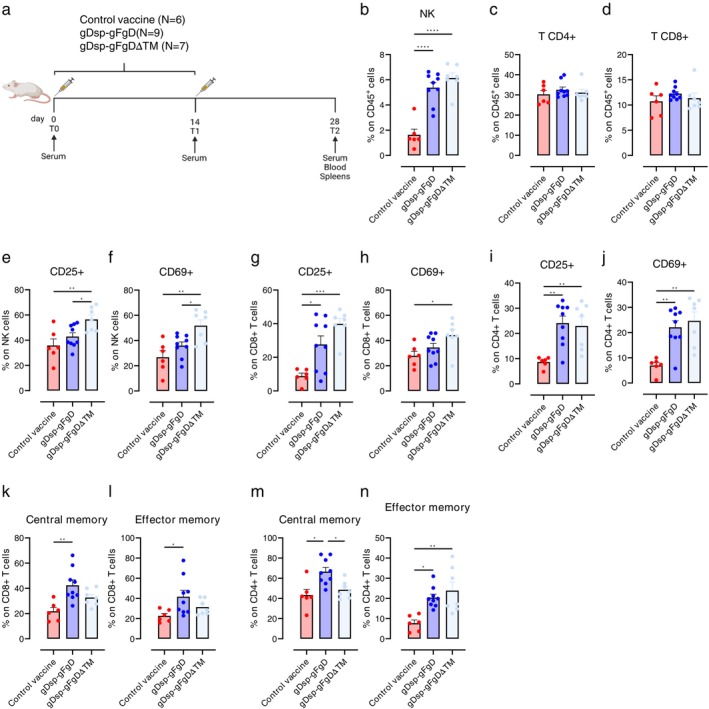
Vaccination with gDsp‐gFgD and gDsp‐gFgDΔTM activates cellular responses in mice. (a) Schematic diagram of the immunisation schedule and sampling timeline. (b–n) FACS analysis of the peripheral blood of mice vaccinated twice with either control vaccine (*N* = 6), gDsp‐gFgD (*N* = 9) and gDsp‐gFgDΔTM (*N* = 7). Frequency of (b) CD45^+^ CD49b^+^ CD3^−^ NK cells, (c) CD45^+^ CD3^+^ CD4^+^ and (d) CD45^+^ CD3^+^ CD8^+^ T cells in the blood of vaccinated mice. FACS analysis of (e, g, i) CD25^+^ and (f, h, j) CD69^+^ activated (e, f) NK, (g, h) CD8^+^ and (i, j) CD4^+^ T cells. FACS analysis of (k, m) central memory CD44^+^ CD62L^+^ and (l, n) effector/effector memory CD44^+^ CD62L^−^ (k, l) CD8^+^ and (m, n) CD4^+^ T lymphocytes. Graphs show the single mice and the means ± SEM of the frequency of cells. Normality was confirmed with the Shapiro–Wilk test. *p < 0.05, **p < 0.01, ***p < 0.001, ****p < 0.0001; One‐Way ANOVA, followed by Tukey's multiple comparison test.

To assess the immunogenic potential of BoHV4 delivering the full‐length or the ΔTM gDsp‐gFgD sequences, BALB/c mice were vaccinated with the two vaccines or with a control BoHV‐4 delivering an unrelated expression cassette for a monkeypox virus glycoprotein (BoHV‐4‐A29, hereafter called control vaccine) [20]. Specifically, mice received two vaccinations, 2 weeks apart, and were sacrificed 2 weeks after the second vaccination (Figure 3a). Blood, sera and spleens were collected for immune response analyses.

FACS analysis of blood revealed a significant increase in NK‐cell percentages in both groups of mice vaccinated with gDsp‐gFgD and gDsp‐gFgDΔTM compared to mice vaccinated with the control vaccine, while no differences were observed in the abundance of CD8^+^ and CD4^+^ T cells (Figure 3b–d). The analysis of the activation markers CD25 and CD69 showed that activated NK and CD4^+^ T cells were significantly enhanced by gDsp‐gFgD vaccine as compared to the control vaccine, and a trend of increase in activated CD8^+^ T cells was observed. gDsp‐gFgDΔTM significantly increased the frequency of activated NK, CD8^+^ and CD4^+^ T cells compared to the control (Figure 3e–j). Moreover, vaccination with gDsp‐gFgD induced the expansion of both central memory CD44^+^ CD62L^+^ and effector/effector memory CD44^+^ CD62L^−^ CD8^+^ and CD4^+^ T cells. gDsp‐gFgDΔTM induced a trend of increase in central memory CD8^+^ and CD4^+^ T cells and in effector/effector memory CD8^+^ T cells, while it significantly enhanced the frequency of effector/effector memory CD4^+^ T lymphocytes (Figure 3k–n). These results provide indication that gDsp‐gFgD and gDsp‐gFgDΔTM are both able to promote cellular immune responses in mice.

### 2.4 Immunisation With gDsp‐gFgD and gDsp‐gFgDΔTM Induces Specific T‐Cell Responses

**FIGURE 4:**
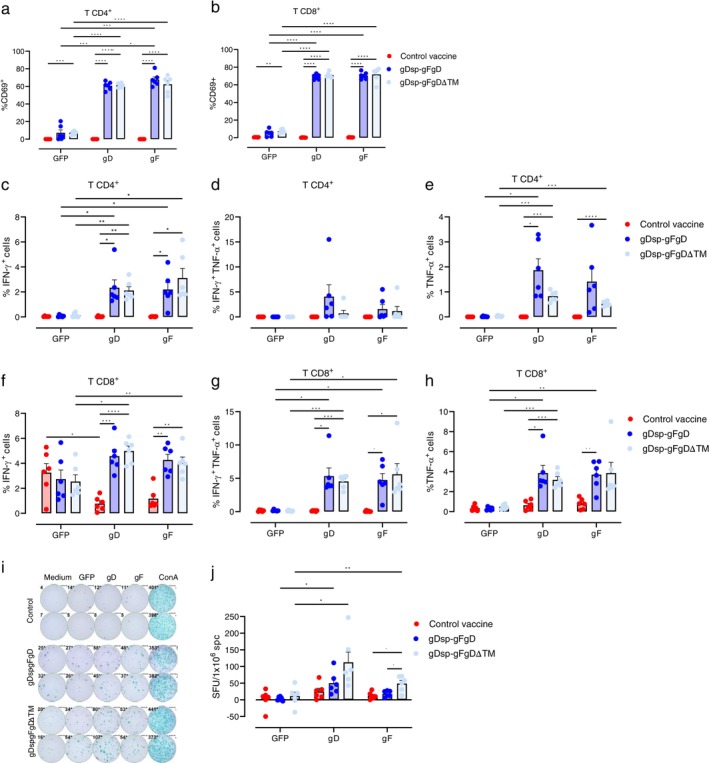
Immunisation with gDsp‐gFgD and gDsp‐gFgDΔTM induces T‐cell responses specific for both gD and gF antigens. The splenocytes from mice vaccinated with either the control vaccine, gDsp‐gFgD or gDsp‐gFgDΔTM were re‐stimulated ex vivo for 48 h with either GFP (negative control), gDΔTM and gFΔTM‐HA antigens (*N* = 6 per group). (a, b) FACS analysis of the frequency of activated CD69^+^ (a) CD4^+^ and (b) CD8^+^ T cells. (c–h) FACS intracellular staining of the frequency of (c) IFN‐γ^+^, (d) IFN‐γ^+^ TNF‐α^+^, (e) TNF‐α^+^ CD4^+^ and (f) IFN‐γ^+^, (g) IFN‐γ^+^ TNF‐α^+^, (h) TNF‐α^+^ CD8^+^ T lymphocytes. (i, j) IFN‐γ ELISpot of splenocytes of control or vaccinated mice, analysed after 48 h in vitro re‐stimulation with or without either GFP (negative control), gDΔTM and gFΔTM‐HA antigens, or with ConA (2 μg/mL) as a positive control. (i) Representative images from 2 mice per group are shown. (j) The graph shows the results from single mice and the means ± SEM of the number of spot forming units (SFU)/10^6^ cells, calculated after the subtraction of the SFU measured in wells where splenocytes were incubated in medium alone. *p < 0.05, **p < 0.01, ***p < 0.001, ****p < 0.0001; Two‐Way ANOVA followed by multiple comparison Tukey's test.

The specificity of T‐cell responses induced by gDsp‐gFgD and gDsp‐gFgDΔTM vaccines was characterised ex vivo through different immune‐based assays using splenocytes from vaccinated mice. In particular, splenocytes were incubated and re‐stimulated with the gDΔTM and gFΔTM‐HA proteins. GFP was used as a control. After 48 h, FACS analysis was performed to analyse the induced T‐cell responses. Re‐stimulation with both gDΔTM and gFΔTM‐HA induced the activation of CD4^+^ and CD8^+^ T cells from mice vaccinated with gDsp‐gFgD and gDsp‐gFgDΔTM, but not from mice immunised with the control vaccine (Figure 4a,b). To characterise the type of T‐cell responses, the intracellular production of interferon (IFN)‐γ and tumour necrosis factor (TNF)‐α was analysed. Immunisation with the control vaccine did not increase the frequency of CD4^+^ or CD8^+^ T cells producing either IFN‐γ or TNF‐α, nor of those producing both cytokines, upon re‐stimulation with gDΔTM and gFΔTM‐HA antigens, confirming that the control vaccine does not induce T‐cell activation. On the contrary, both gDsp‐gFgD and gDsp‐gFgDΔTM vaccines induced an increase in CD4^+^ and CD8^+^ T cells producing either IFN‐γ or TNF‐α, or both cytokines, upon re‐stimulation with gDΔTM and gFΔTM‐HA antigens, but not with the unrelated GFP protein (Figure 4c–h). To confirm that gDsp‐gFgD and gDsp‐gFgDΔTM vaccines induce Th1‐type immune responses, an IFN‐γ ELISpot assay was performed on splenocytes from vaccinated mice re‐stimulated for 48 h with either GFP, gDΔTM and gFΔTM‐HA proteins. Cells cultured in medium or stimulated with concanavalin A (ConA) were used as additional negative and positive controls, respectively. As shown by the representative images and the graph, higher numbers of IFN‐γ‐producing cells were observed in splenocytes from mice vaccinated with gDsp‐gFgD and gDsp‐gFgDΔTM upon re‐stimulation with both gDΔTM and gFΔTM‐HA antigens, but not with the GFP protein (Figure 4i,j), further confirming that both gDsp‐gFgD and gDsp‐gFgDΔTM vaccines are able to induce Th1‐type 1 immune responses specific for gD and gF antigens in both CD4^+^ and CD8^+^ T lymphocytes.

### 2.5 Immunisation With gDsp‐gFgD and gDsp‐gFgDΔTM Vaccines Induces T Cell‐Mediated Cytotoxicity Against Cells Expressing gD and gF Antigens

**FIGURE 5:**
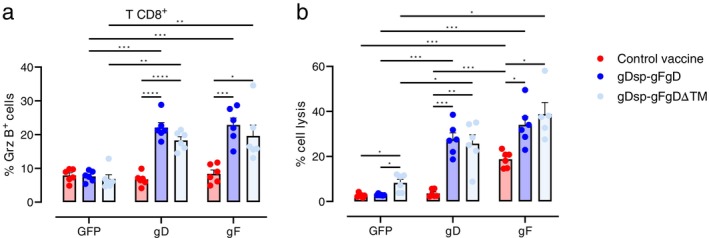
gDsp‐gFgD and gDsp‐gFgDΔTM vaccines induce cytotoxic T cells specific for gD and gF. (a) The splenocytes from mice vaccinated with either the control vaccine, gDsp‐gFgD or gDsp‐gFgDΔTM were re‐stimulated for 48 h with either GFP (negative control), gDΔTM and gFΔTM‐HA antigens (*N* = 6 per group), and the frequency of GrzB^+^ CD8^+^ T cells was analysed by FACS. (b) 3T3‐kB cells were transfected with plasmids coding for GFP, gD and gF (pEGFP‐C1, pgD‐WPRE and pgF) proteins, stained with CFSE and incubated with splenocytes from control vaccine, gDsp‐gFgD or gDsp‐gFgDΔTM‐vaccinated mice at a 200:1 ratio for 48 h. Cell death was assessed by FACS following 7‐AAD staining. Graphs show values from individual mice and mean ± SEM of the percentages of cell lysis. *p < 0.05, **p < 0.01, ***p < 0.001, ****p < 0.0001; Two‐Way ANOVA followed by multiple comparison Tukey's test.

To verify whether a specific cytotoxic T‐cell response is induced by the vaccination with gDsp‐gFgD and gDsp‐gFgDΔTM, splenocytes from vaccinated mice were re‐stimulated in vitro with the gDΔTM and gFΔTM‐HA antigens, or GFP as a control. The production of granzyme B (GrzB) by CD8^+^ T lymphocytes was measured by FACS 48 h later. As shown in Figure 5a, incubation with both gDΔTM and gFΔTM‐HA proteins, but not with GFP, significantly increased the frequency of GrzB^+^ cytotoxic T cells in splenocytes collected from mice vaccinated with gDsp‐gFgD and gDsp‐gFgDΔTM as compared to those from mice that received the control vaccine. To verify the functionality of gD‐ and gF‐specific cytotoxic T cells induced by the two vaccines, an in vitro cytotoxicity assay was performed. Specifically, NIH/3T3 BALB/c mouse fibroblasts overexpressing the BALB/c major histocompatibility molecule (MHC) H‐2Kd and the co‐stimulatory molecule B7.1 (3T3/kB) were transiently transfected with plasmids expressing the gF, gD (pgF and pgD‐WPRE, respectively) or control GFP (pEGFP‐C1) proteins. After 24 h, transfected cells were stained with CFSE and incubated for 48 h with splenocytes from syngeneic vaccinated mice, then target cell lysis was analysed by FACS. Although a low but significant increase in the cytotoxicity of gF^+^ as compared to GFP^+^ or gD^+^ target cells was observed with splenocytes from mice immunised with the control vaccine, as well as a slight increase in the killing of GFP^+^ 3T3/kB cells was induced by T lymphocytes from mice vaccinated with gDsp‐gFgDΔTM, splenocytes from both gDsp‐gFgD and gDsp‐gFgDΔTM vaccinated mice induced a significantly higher cytotoxicity of 3T3/kB cells expressing gD or gF as compared to those from mice treated with the control vaccine (Figure 5b).

Overall, these results demonstrate that both gDsp‐gFgD and gDsp‐gFgDΔTM vaccines induce a strong and specific cytotoxic T‐cell response against both gD and gF expressing cells, supporting their further development for the prevention of BoHV‐1 and BRV1 infections in cattle.

### 2.6 Immunisation With BoHV‐4‐A‐CMV‐gDsp‐gF‐gD‐ΔTK and BoHV‐4‐A‐CMV‐gDsp‐gF‐gDΔTM‐ΔTK Vaccines Induces Specific Humoral Responses

**FIGURE 6:**
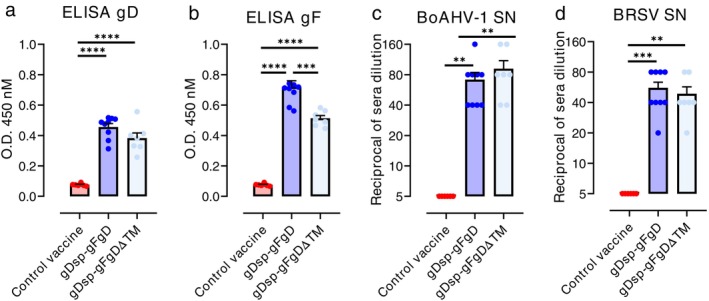
Serum total IgGs and neutralising antibody response in mice following immunisation. (a) Sera from groups of mice immunised with BoHV‐4‐A‐CMV‐A29‐ΔTK (Control vaccine), BoHV‐4‐A‐CMV‐gDsp‐gF‐gD‐ΔTK (gFgD) and BoHV‐4‐A‐CMV‐gDsp‐gF‐gDΔTM‐ΔTK (gF‐gDΔTM) were assessed by ELISA for antibody response against BoHV‐1 gD (a; ELISA gD) and BRSV gF (b; ELISA gF). Data are expressed as individual values obtained for each animal, using a 1:10 dilution of serum and expressed as the optical density (O.D.) at 450 nm. The same sera were assessed for neutralising antibody response; SN antibodies were expressed as the reciprocal of the highest dilution of the serum that inhibited the development of BoHV‐1‐induced CPE (BoHV‐1 SN; c) or BRSV‐induced CPE (BRSV SN; d) in AUBEK cells. *p < 0.05; **p < 0.01; ***p < 0.001; ****p < 0.0001, as measured by ONE‐way multiple comparisons ANOVA with Tukey's post‐test.

BALB/c mice were vaccinated twice with BoHV‐4‐A‐CMV‐gDsp‐gF‐gD‐ΔTK and BoHV‐4‐A‐CMV‐gDsp‐gF‐gDΔTM‐ΔTK or with a control BoHV‐4 delivering an expression cassette for an unrelated monkeypox virus glycoprotein antigen, to ensure the detection of a specific response. Blood samples were collected from mice at sacrifice, 2 weeks after the second vaccination, for the assessment of antibody response against BoHV‐1 and BRSV. ELISA plates were coated with gDΔTM and gFΔTM‐HA proteins. Sera from both gDsp‐gFgD and gDsp‐gFgDΔTM‐vaccinated animals showed a specific total antibody response when tested against gDΔTM or gFΔTM‐HA proteins, whereas control animals showed no detectable antibody response (Figure 6a,b). Next, serum neutralisation assay (SN) was performed with serial two‐fold dilutions of each serum and mixed with virus suspension containing BoHV‐1 or BRSV. Permissive AUBEK cells were then added to each well and the plates incubated for 3 days. CPE was detected by microscopy and classical crystal violet staining of the cell monolayer. High level of specific BoHV‐1 and BRSV SN antibodies were found in both gDsp‐gFgD and gDsp‐gFgDΔTM vaccinated animals, compared to the control group of animals, which lacked neutralising activity (Figure 6c,d). In both vaccinated groups, neutralising antibody titers against BoHV‐1 ranged from 1:40 to 1:160, while those against BRSV ranged from 1:20 to 1:80. Mean neutralising titers were similar across vaccinated groups: against BoHV‐1, the mean neutralising titers were 71.1 ± 38.9 for gDsp‐gF‐gD and 91.4 ± 50.1 for the secreted variant gDsp‐gF‐gDΔTM. Against BRSV, gDsp‐gF‐gD induced a mean titer of 55.6 ± 24.0, whereas gDsp‐gF‐gDΔTM elicited a mean of 48.6 ± 22.7.

## 3 Discussion

In this study, we developed and characterised a novel bivalent vaccine based on BoHV‐4 engineered to express chimerized antigens from BoHV‐1 and BRSV, two major viral pathogens contributing to BRD.

The development of effective vaccines for livestock diseases such as BRD contributes not only to animal health and welfare but also to reducing antibiotic use, mitigating antimicrobial resistance (AMR) and ensuring sustainable food systems. In this context, multivalent vaccines that limit co‐infections and disease severity can play a critical role in integrated disease management strategies. Furthermore, BRSV and BoHV‐1, share similarities with human pathogens, making them valuable comparative models for vaccine development and immunological studies [21]. Current approach for BRD prevention relies on multivalent vaccines, typically including modified live BoHV‐1 and BRSV strains [11]. However, protection is often incomplete due to limited immunogenicity, short duration of immunity, and interference from colostrum‐derived antibodies, highlighting the need for new strategies that can provide more consistent and effective protection.

Previous studies investigating the immunogenicity of various *alphaherpesvirus* gD, including BoHV‐1, demonstrated its capacity to induce robust virus neutralising antibodies and strong cell‐mediated immune responses, as well as protection from clinical disease in target species [3]. Similarly, the BRSV gF has emerged as a promising target for vaccine development due to its critical role in viral entry, high degree of conservation across isolates, and the presence of multiple neutralising epitopes [6]. Given the high immunogenic potential of these viral antigens, BRSV gF and BoHV‐1 gD were selected for the generation of gDsp‐gF‐gD chimeric peptide. Chimeric peptides represent a valuable strategy for the expression of multiple antigens in the context of polyvalent vaccine development, offering several advantages over other co‐expression methods. For instance, the internal ribosome entry site (IRES) of the encephalomyocarditis virus can recruit ribosomes to an internal region of the mRNA to initiate translation, thereby enabling the development of bicistronic or polycistronic vectors. However, reduced expression of the cistron placed downstream of the IRES frequently occurs, resulting in inefficient immunisation against one or more antigens. Furthermore, the employment of IRES elements result in the generation of unusually long ORFs which may negatively affect adaptability to the delivery system or expression [14, 19, 22]. Conversely, chimeric peptides ensure balanced co‐expression of multiple antigens while minimising ORF length. Moreover, the combination of multiple antigens from different pathogens in a single chimeric peptide reduces the time and cost of downstream processes such as purification and formulation, ultimately improving the efficiency of application [23, 24, 25]. However, chimeric peptides may exhibit altered folding patterns that could affect immunogenicity by masking or modifying critical epitopes. Therefore, the preservation of immunogenicity and functional activity should be experimentally verified [19].

The gDsp‐gF‐gD construct was further modified to produce a transmembrane‐deleted variant (gDsp‐gF‐gDΔTM), enabling secretion and allowing for different antigen‐processing and presentation by the immune system. Antigens secreted by viral vector transduced cells are captured by APC for exogenous processing pathway, loaded onto MHC class II molecules and presented to CD4^+^ helper T cells, ultimately resulting in B cells stimulation and antibody production. In line with this mechanism, previous investigations have demonstrated that secreted forms of antigens can elicit stronger humoral immune responses than their membrane‐bound counterparts [26, 27]. Moreover, secreted antigens may also elicit a stronger cell‐mediated immune response being more accessible to host immune system than membrane‐bound antigens [27].

Both the membrane‐bound (gDsp–gF–gD) and the secreted (gDsp–gF–gDΔTM) chimeric peptides were delivered using the same viral vector, resulting in endogenous antigen expression within infected host cells. Viral vector–mediated delivery promotes intracellular synthesis of the antigen, thereby favouring processing through the cytoplasmic antigen‐processing machinery. Following translation in the cytosol, both constructs are likely subjected to proteasomal degradation, generating peptide fragments that are subsequently transported into the endoplasmic reticulum via the transporter associated with antigen‐processing (TAP). These peptides can then be loaded onto MHC class I molecules for presentation to CD8^+^ T cells.

Although the two constructs differ in their membrane association and secretion profiles, these structural differences may have limited impact on early intracellular processing events driven by viral vector expression. In addition to MHC class I presentation, a proportion of the expressed antigen may also enter the MHC class II pathway through autophagy or endosomal trafficking, enabling activation of CD4^+^ T cells. The shared intracellular route of antigen expression and processing likely results in overlapping peptide repertoires being presented to the immune system. Consequently, this common cytoplasmic processing and presentation pathway may explain the comparable immune responses elicited by both the membrane‐bound and secreted chimeric peptides, suggesting that the mode of antigen delivery and intracellular handling plays a more decisive role than antigen localisation in shaping the overall immunogenicity.

While both gDsp‐gF‐gD and gDsp‐gF‐gDΔTM constructs could represent valuable tools for immunisation purposes, potentially adaptable to different delivery systems such as adjuvated protein vaccines, self‐assembling nanoparticle or nucleic acid vaccines (DNA or mRNA) [28, 29], a BoAHV‐4‐based vector was selected because of its proven efficacy as an antigen delivery system in both cattle and BALB/c mice model [30, 31].

BoHV‐4 offers several advantages as a viral vector, including a non‐pathogenic profile, broad host cell tropism, and genomic capacity for multiple transgene insertions. Unlike other herpesviruses, BoHV‐4 does not appear to interfere with the bovine immune system, making it a safe and versatile delivery system [32]. One limitation of viral vector vaccines is that they often elicit vector‐specific neutralising antibodies, potentially rendering booster immunisations less effective or even ineffective. Similarly, the efficacy of viral vectors such as those based on adenovirus and adeno‐associated viruses is limited by a high prevalence in the human population of antibodies induced by naturally circulating virus serotypes which may neutralise the vaccine and inhibit its capability to infect the host cells [33]. On the contrary, BoHV‐4 does not induce neutralising antibodies in cattle [15] nor in mice [34], allowing repeated administrations.

Our results demonstrate that BoHV‐4 is an effective viral vector for delivering multiple antigens and inducing robust antigen‐specific T and B cell responses in a murine model, thus providing a promising platform for the development of multivalent veterinary vaccines.

In this paper, a proof‐of‐concept study aimed at assessing the immunogenicity of two bivalent hybrid vaccines targeting both BoHV‐1 and BRSV1 was performed. Female BALB/c mice were vaccinated twice with BoHV‐4‐A‐CMV‐gDsp‐gF‐gD‐ΔTK and BoHV‐4‐A‐CMV‐gDsp‐gF‐gDΔTM‐ΔTK or with a control BoHV‐4 delivering an unrelated antigen for the assessment of both humoral and cellular immune responses. ELISA of mouse sera indicated that both gDsp‐gFgD and gDsp‐gFgDΔTM vaccinated animals showed strong total antibody responses against both BoHV‐1 gD and BRSV gF, demonstrating that vaccination elicited a specific immune response. Neutralising antibodies against BoHV‐1 are important for protection from disease and viral shedding mitigation [35, 36]. Similarly, BRSV neutralising antibodies (maternally derived or due to previous infection) correlate with protection against severe disease and viral load reduction in the lower respiratory tract [37, 38]. In order to assess neutralising antibodies production in vaccinated mice, a SN assay was performed. High levels of specific BoHV‐1 and BRSV sero‐neutralising antibodies were found in both gDsp‐gFgD and gDsp‐gFgDΔTM vaccinated groups. Interestingly, vaccination induced comparable SN titers against BoHV‐1 and BRSV, with no marked differences between groups immunised with membrane‐bound or secreted antigens.

Although neutralising antibodies play a significant role in protection, they are insufficient to fully prevent disease or establishment of latency in BoHV‐1 infection. Effective control requires a strong cell‐mediated immune response critical for limiting viral replication and clearing infected cells [39, 40]. Similarly, cell mediated immunity plays a critical role in protection and recovery from BRSV infection [37].

Vaccination of BALB/c mice elicited a significant expansion and activation of NK cells. This observation is in agreement with previous studies showing that NK cells can be activated after vaccination in humans and mice. In humans, NK‐cell responses to vaccine antigens such as diphtheria and tetanus toxoids, or whole cell pertussis, are enhanced during recall responses and are thought to depend largely on IL‐2 released by antigen‐specific CD4^+^ T lymphocytes [41, 42]. Influenza vaccination has likewise been shown to increase NK‐cell responsiveness, including enhanced IFN‐γ production, upregulation of activation markers, and proliferation of less differentiated NK‐cell subsets [43, 44]. In murine models, influenza vaccination also recruits and activates NK cells in draining lymph nodes through type I IFN‐dependent signals, indicating that innate cell‐derived cytokines are central to this process [45]. Moreover, Bacillus Calmette‐Guérin (BCG) vaccination induces robust IFN‐γ‐producing NK‐cell responses in infants, further supporting the view that vaccination can amplify NK‐cell activity through coordinated innate and adaptive cytokine networks [46]. Collectively, these data suggest that the NK‐cell expansion observed here may result from the combined action of T‐cell‐derived IL‐2 and APC‐derived cytokines such as IL‐12, IL‐15, IL‐18 and type I IFNs, which together promote NK‐cell proliferation and activation [41]. However, in this study, the effect of vaccination on NK‐cell function was not assessed; therefore, the actual contribution of the observed increase in NK‐cell frequency and activation marker expression to protective immunity cannot be determined.

Vaccination with gDsp‐gFgD and gDsp‐gFgDΔTM vaccines was able to induce the expansion of activated CD4^+^ and CD8^+^ T lymphocytes as compared to mice immunised with a control BoHV‐4 vaccine. Accordingly, an expansion of central memory and effector/effector memory T lymphocytes was induced. By characterising more deeply the immune response induced by re‐stimulation of T lymphocytes with either the gD or gF antigens, we demonstrated that both vaccines could activate antigen‐specific T cells and induce a Th1‐type immune response, with the expansion of gD and gF‐specific CD4^+^ and CD8^+^ T cells producing IFN‐γ and/or TNF‐α. Of note, a higher proportion of cells co‐expressing IFN‐γ and TNF‐α compared to those expressing either cytokine alone was observed. This likely reflects the enrichment of highly activated, polyfunctional effector cells. Indeed, in T cells, cytokine production is often coordinated, such that strongly stimulated cells simultaneously produce multiple pro‐inflammatory cytokines. Accordingly, multiple vaccination studies have demonstrated the induction of polyfunctional CD4^+^ or CD8^+^ T cells co‐producing IFN‐γ and TNF‐α, which more reliably correlate with the extent of vaccine‐induced protection than those producing IFN‐γ alone [47]. Moreover, both vaccines induced the expansion of antigen‐specific cytotoxic T cells producing granzyme b, which were capable of recognising and responding to cells expressing the vaccine antigens. These findings support the cytotoxic potential of vaccine‐induced T cells, a critical component of protective immunity against intracellular pathogens such as BoHV‐1 and BRSV. While most currently licenced vaccines primarily induce humoral responses, our platform emphasises the importance of cell‐mediated immunity, particularly for pathogens capable of establishing latency (BoHV‐1) or suppressing host immune responses (BRSV).

Although mice are not the most representative animal model for BoHV‐1 and BRSV, they remain a cornerstone of preclinical research because of their utility in characterising immune mechanisms relevant to vaccine and immunotherapeutic development. Neither BoHV1 nor BRSV naturally infect mice, and murine infection does not fully recapitulate the clinical course, viral tropism, or pathogenesis observed in cattle. Consequently, murine models have clear limitations in evaluating disease severity, viral transmission, and host‐pathogen interactions at the respiratory mucosal level in the natural host [48]. Despite these constraints, mouse models are widely used for early immunogenicity screening and mechanistic studies. In the context of BRSV, murine models have been instrumental in dissecting innate and adaptive immune responses, including the roles of CD4^+^ and CD8^+^ T cells, antibody isotype profiles, and cytokine polarisation following vaccination or antigen exposure. Studies using RSV and BRSV antigens in mice have shown that vaccine‐induced immune signatures—such as Th1/Th2 balance, neutralising antibody titers, and memory T‐cell development—can inform downstream selection of vaccine formulations, adjuvants and delivery routes. Although protection data in mice do not always translate directly to cattle, these immunological readouts are highly valuable for comparative evaluation of candidates before testing in more complex and costly ruminant models [48]. Similarly, for BoHV‐1, murine studies have been extensively used to evaluate vaccine immunogenicity, adjuvant effects and antigen design. Multiple studies have demonstrated that mice immunised with BoHV‐1 DNA vaccines, recombinant viruses, or subunit antigens mount measurable humoral and cellular immune responses that parallel, at least qualitatively, those observed in cattle. For example, murine models have been used to assess IgG subclass skewing, IFN‐γ production and cytotoxic T‐lymphocyte activation following vaccination with BoHV‐1 glycoproteins, providing early evidence of Th1‐biased immunity that is desirable for viral control. These parameters are particularly useful for ranking vaccine constructs and adjuvants prior to validation in bovine infection or challenge models. Importantly, mouse models offer practical and experimental advantages that are critical for preclinical decision‐making. Their low cost, genetic homogeneity, availability of immunological reagents, and compatibility with transgenic and knockout approaches make mice uniquely suited for hypothesis‐driven studies of immune mechanisms and correlates of protection. As a result, murine immunogenicity data are routinely used to de‐risk development pipelines by identifying non‐performing candidates early and prioritising those with favourable immune profiles for advancement into target‐species studies [49]. In summary, while mice are not the optimal model for reproducing BoHV‐1 or BRSV disease in cattle, preclinical immunological data generated in mice play a pivotal role in guiding vaccine development. When interpreted within their biological limitations and integrated with data from bovine or other relevant models, murine studies provide essential evidence to support rational decision‐making, candidate down‐selection, and optimisation strategies in the early stages of preclinical research [50].

This study establishes a critical preclinical proof‐of‐concept supporting the rational progression toward in vivo challenge experiments and eventual vaccine development in cattle, the natural host of the pathogens. Although no challenging study was performed, the data generated are essential to determine whether advancement to this ethically, logistically, and economically demanding stage is scientifically justified. We demonstrate that a single chimeric peptide immunogen can elicit both humoral and cellular adaptive immune responses against two distinct pathogens, highlighting the potential of a dual‐target strategy to enhance immune coverage while limiting formulation complexity. Given the substantial costs and ethical constraints associated with bovine challenge studies, such experiments require robust preliminary evidence. Accordingly, this work prioritises the identification of key immunological correlates of protection, including neutralising antibodies and cell‐mediated immunity, generated in a laboratory animal model. These findings provide the necessary foundation for informed decisions regarding further development. Overall, this study represents an essential intermediate step in a stepwise vaccine development pathway and supports the broader applicability of chimeric immunogens as a platform for targeting multiple pathogens across different animal species.

Ultimately, such approaches may contribute to improved disease control of very similar viruses in human beings. hRSV is a predominant etiological agent of acute respiratory infections (ARI) in paediatric populations under 5 years of age [51]. However, its clinical relevance extends to geriatric cohorts, where age‐related immunosenescence contributes to increased susceptibility and severity of disease. Globally, hRSV‐related morbidity in older adults results in an estimated 14 000 in‐hospital fatalities annually [52]. The associated healthcare burden is substantial, with cost analyses from the United States reporting per‐patient hospitalisation expenses ranging from $8241 to $23 194 annually for patients aged ≥ 65 years [53]. Herpes zoster (HZ), commonly referred to as shingles, similarly exhibits increased incidence in older adults due to the reactivation of latent VZV residing in the dorsal root ganglia following primary infection—typically acquired during childhood as varicella (chickenpox). Approximately 95% of adults harbour latent VZV, placing them at risk for HZ reactivation [54]. The clinical manifestation of HZ involves a painful vesicular dermatomal eruption, which may progress to post‐herpetic neuralgia (PHN), a chronic neuropathic pain syndrome persisting for months to years [54]. The economic impact of HZ and PHN is considerable, with direct medical costs estimated at €271.21 million (≈$301.04 million) in the United Kingdom in 2015 and $2.6 billion in the United States in 2020 [54].

## 4 Methods

### 4.1 Cells and Viruses

Murine NIH/3T3 fibroblasts stably expressing H‐2Kd and B7.1 (3T3/kB) were cultured in DMEM (ThermoFisher Scientific) supplemented with 20% FBS [34].

HEK (human embryonic kidney cells) 293 T (ATCC: CRL‐11268), BEK (bovine embryo kidney; Istituto Zooprofilattico Sperimentale, Brescia, Italy; BS CL‐94), BEK cells expressing *cre* recombinase [19], MDBK (Madin–Darby bovine kidney cells; ATCC: CRL‐6071) and AU‐BEK (Auburn University‐Bovine Embryonic Kidney, RRID:CVCL_6533) were grown in complete Eagle's minimal essential medium (Euroclone, cEMEM: 1 mM of sodium pyruvate, 2 mM of L‐glutamine, 100 IU/mL of penicillin, 100 μg/mL of streptomycin and 0.25 μg/mL of amphotericin B), supplemented with 10% FBS and incubated at 37°C, 5% CO_2_. All the supplements were purchased from Gibco.

BoHV‐1 (Strain New York, GenBank accession number NC_001847.1) was propagated by infecting confluent monolayers of BEK or MDBK at a multiplicity of infection (M.O.I.) of 0.1 50% tissue culture infectious doses (TCID_50_) per cell and maintained in cEMEM with 2% FBS for 2 h. The medium was then removed and replaced by fresh cEMEM containing 10% FBS. When cytopathic effect (CPE) affected the majority of the cell monolayer (approximately 48 h post‐infection), the virus was prepared by freezing and thawing cells three times and pelleting the virions through a 30% sucrose gradient [55]. Virus pellets were resuspended in cold cEMEM without FBS. TCID_50_ were determined in BEK or MDBK cells by limiting dilution.

BRSV (Strain 375, ATCC: VR‐1339) was propagated in AU‐BEK cells, modifying the protocol described for MDBK infection [56]; the highly cell‐associated virus particles were released by freezing and thawing the infected monolayers and titrated classically in AU‐BEK cells.

### 4.2 Plasmids Generation

gDsp‐gF‐gD nucleotidic sequence was chemically synthesised (Genescript) and sub‐cloned in a BoHV‐4 shuttle vector with expression cassettes flanked by BoHV‐4 TK sequences, named pINT2‐EGFP and previously described [32]; gDsp‐gF‐gD and the shuttle vector were cut with NheI and SmaI restriction enzymes, generating pINT2‐CMV‐gDsp‐gF‐gD.

pINT2‐CMV‐gDsp‐gF‐gDΔTM was obtained by amplifying gDsp‐gF‐gDΔTM from pINT2‐CMV‐gDsp‐gF‐gD, using gDsp‐gF‐deltaTm‐sense and gDsp‐gF/gDdeltaTm‐antisense couple of primers (see Table 1) that attached a NheI site at the 5′ end of the ORF and SmaI site at the carboxyterminal to the nucleotidic sequence. After the restriction digestion with these two enzymes, the amplicon was subcloned into pINT2‐EGFP, cut with the same enzymes, to finally generate pINT2‐CMV‐gDsp‐gF‐gDΔTM.

**TABLE 1:** List of primers used in this work.

Primer name	Sequence 5′‐3′
gF‐deltaTm‐HA‐sense	CCCGCTAGCCCACCATGGCCACCACCGCCATGTCGCATGATCATC
gF‐deltaTm‐HA‐antisense	CCCCCCGGGTTAGTGCGTAGTCGGGCACGTCGTAGGGGTAGTTGGTGGTGCTCTTGCCCACGTCCACGCT
gDsp‐gF‐deltaTm‐sense	CCCGCTAGCCCACCATGCAGGGCCCCACCCTGG
gDsp‐gF/gDdeltaTm‐anti	AAACCCGGGTCAGCTCACGGGCACGGCGTC
NheI‐gDdeltaTm‐sense	CCCGCTAGCCCACCATGCAAGGGCCGACATTGGCC
KpnI‐gDdeltaTm‐antisense	CCCGGTACCTCACGGCACGGCGTCGGGGGCCGC

The PCR amplification reaction was carried out in a final volume of 50 μL, containing 20 mM Tris–hydrochloride pH 8.8, 2 mM MgSO_4_, 10 mM KCl, 10 mM (NH_4_)_2_SO_4_, 0.1 mg/mL BSA, 0.1% (v/v) Triton X‐100, 5% dimethyl sulfoxide (DMSO), 0.2 mM deoxynucleotide triphosphate, and 0.25 μM of each primer. 1 U of Phusion high fidelity DNA polymerase (ThermoFisher Scientific) was used to amplify 100 ng of template DNA over 35 repeated cycles, consisting in 1 min of denaturation at 94°C, 1 min of annealing at 62°C and 2 min 30 s of elongation at 72°C.

The nucleotide sequence coding for the BRSV gF ORF was also chemically synthesised and labelled with the hemagglutinin (HA) tag via a PCR reaction, using gF‐deltaTm‐HA‐sense and gF‐deltaTm‐HA‐antisense couple of primers (see Table 1), providing a NheI site at the 5′ of the ORF and the HA tag, followed by a SmaI site at the carboxyterminal. The PCR amplification was carried on as described above, varying the amplification parameters as follows: according to the primers melting temperature: 1 min of denaturation at 94°C, 1 min of annealing at 60°C and 1 min 40 s of elongation at 72°C, repeated for 35 cycles. The amplicon was cut with NheI/SmaI and inserted in pINT2‐EGFP [32], cut with the same enzymes, to generate pINT2‐gFΔTM‐HA.

The secreted form of gD was generated by PCR amplification from pgD‐WPRE [55], using NheI‐gDdeltaTm‐sense and KpnI‐gDdeltaTm‐antisense couple of primers (described in Table 1); the amplicon was then restriction digested with NheI/KpnI and subcloned in pINT2‐EGFP, cut with the same enzymes, to obtain pINT2‐gDΔTM. The amplification parameters used were 1 min of denaturation at 94°C, 1 min of annealing at 55°C and 1 min at 72°C, repeated for 35 cycles.

### 4.3 Transient Transfection and Syncytia Formation

HEK293T cells were seeded into 25 cm^2^ flasks (1 × 10^6^ cells/flask) and transfected with pINT2‐CMV‐gDsp‐gF‐gD, pINT2‐CMV‐gDsp‐gF‐gDΔTM or pEGFP‐C1 (mock control, Clontech) using polyethyleneimine (PEI) transfection reagent (Polysciences Inc.). DNA was mixed with PEI in a ratio 1:2.5 DNA:PEI in 500 μL of serum‐free Dulbecco's modified essential medium (DMEM) with high glucose (Euroclone) and incubated 15 min at room temperature, 4× volumes of serum‐free medium were added and the transfection solution was transferred onto the cells monolayer for 6 h at 37°C with 5% CO_2_, in a humidified incubator. HEK293T cells were also transfected with pINT2‐CMV‐gDsp‐gF‐gD, pINT2‐CMV‐gDsp‐gF‐gDΔTM, pINT2‐gFΔTM‐HA, pINT2‐gDΔTM or pEGFP‐C1 to study protein secretion. To this scope, after the 6 h of incubation, the transfection mixture was replaced by 1:1 DMEM/F12 and after 48 h of incubation at 37°C, 5% CO_2_, the cell supernatant was collected, clarified and stored at −80°C. Twenty‐four hours after transient pINT2‐CMV‐gDsp‐gF‐gD or pINT2‐CMV‐gDsp‐gF‐gDΔTM transfection, HEK cells were also observed for syncytia formation, by inverted fluorescence microscope (Zeiss‐Axiovert‐S100), and pictures were acquired by digital camera (Zeiss‐Axiocam‐MRC).

### 4.4 Immunoblotting

Protein cell extracts were obtained from pINT2‐CMV‐gDsp‐gF‐gD, pINT2‐CMV‐gDsp‐gF‐gDΔTM, pINT2‐gFΔTM‐HA, pINT2‐gDΔTM or pEGFP‐C1 transfected HEK 293T cells by adding 100 μL of cell extraction buffer (50 mM Tris–HCl, 150 mM NaCl and 1% NP‐40; pH 8). BEK cells were also infected with 0.5 M.O.I. of BoHV‐4‐A‐CMV‐gDsp‐gF‐gDΔTK and BoHV‐4‐A‐CMV‐gDsp‐gF‐gDΔTM‐ΔTK or left uninfected and analysed by western blotting. After BCA total protein quantification (Pierce BCA Protein Assay kit, Thermoscientific), cell extracts containing various amounts of total protein were electrophoresed through 10% SDS‐PAGE. Membranes were probed with primary anti‐BoHV‐1 gD monoclonal antibody (clone 1B8‐F11; VRMD), diluted 1:15000 or with a mouse monoclonal antibody anti‐HA tag (G036, Abm Inc) diluted 1:10000 (only for gFΔTM‐HA) and with horseradish peroxidase‐labelled secondary anti‐mouse immunoglobulin (A9044, Sigma‐Aldrick, Merck), diluted at 1:15000. The signals were visualised using enhanced chemiluminescence (Clarity Max Western ECL substrate, Bio‐Rad). Cell supernatants, obtained from HEK 293T cells transfected with pINT2‐CMV‐gDsp‐gF‐gD, pINT2‐CMV‐gDsp‐gF‐gDΔTM, pINT2‐gFΔTM‐HA, pINT2‐gDΔTM or pEGFP‐C1, were collected after 48 h in serum‐free medium and analysed through 10% SDS–PAGE gel electrophoresis.

### 4.5 Bacterial Artificial Chromosome (BAC) Recombineering

BAC recombineering was carried out as previously described [19], in SW102 *E. coli* , containing pBAC‐BoHV‐4‐A‐TK‐KanaGalK‐TK, re‐targeting the PvuI linearized pINT2‐CMV‐gDsp‐gF‐gD or pINT2‐CMV‐gDsp‐gF‐gDΔTM into the BoHV‐4 TK locus, containing the Kana‐GalK selector cassette to obtain pBAC‐BoHV‐4‐A‐CMV‐gDsp‐gF‐gDΔTK and pBAC‐BoHV‐4‐A‐CMV‐gDsp‐gF‐gDΔTM‐ΔTK, respectively. After extraction and purification, BAC‐DNA was analysed through HindIII restriction enzyme digestion (Fermentas by Thermoscientific) and 1% agarose gel electrophoresis. Detailed protocols for recombineering are available at the recombineering website (https://redrecombineering.ncifcrf.gov/).

### 4.6 Cell Culture Electroporation and Recombinant Virus Reconstitution, Production and Titration

BEK or BEKcre cells were maintained as a monolayer with cEMEM + 10% FBS. BAC‐DNA (~5 μg) was electroporated in 600 μL DMEM (Euroclone) without serum (Bio‐Rad Gene pulser Xcell, 270 V, 1500 μF, 4‐mm gap cuvettes) into BEK and BEKcre cells. Cells were grown until the appearance of CPE. The virus was then harvested by freezing, thawing cells three times, and tittering the supernatant in BEK cells. The recombinant BoHV‐4s were propagated by infecting confluent monolayers of BEK cells at a multiplicity of infection (M.O.I.) of 0.5 TCID_50_. When most of the cell monolayers displayed the CPE (~48–72 h post infection), the virus was harvested by freezing and thawing cells three times and pelleting the virions through a 30% sucrose cushion. Virus pellets were then resuspended in cold DMEM without FBS. The TCID_50_ dose was determined on BEK cells by limiting dilution.

### 4.7 Virus Growth Curves

BEK cells were infected with BoHV‐4‐A, BoHV‐4‐A‐CMV‐gDsp‐gF‐gDΔTK and BoHV‐4‐A‐CMV‐gDsp‐gF‐gDΔTM‐ΔTK at a M.O.I. of 0.1 and incubated at 37°C for 3 h. Infected cells were then washed with serum‐free cEMEM and finally overlaid with cEMEM + 10% FBS. The supernatants were harvested at daily time points, and the amount of infectious virus was determined by limiting dilution on BEK cells. Viral titre at each time point is reported as the average of triplicate measurements ± standard errors.

### 4.8 Immunofluorescence (IF) Staining and Cytofluorimetric Analyses of Transfected Cells

Sub‐confluent monolayers of HEK 293T cells were transfected with pINT2‐CMV‐gDsp‐gF‐gD, pINT2‐CMV‐gDsp‐gF‐gDΔTM or left un‐transfected. Twenty‐four hours after the transfection, the cells were fixed with 4% paraformaldehyde for 10 min, washed three times with Phosphate Buffer Saline (PBS) and then blocked and permeabilized with PBS containing 0.1% Triton‐X 100, 1% Bovine Serum Albumin (BSA, Merck) and 10% FBS for 1 h. Subsequently, the cells were incubated overnight at 4°C with anti BoHV‐1 gD monoclonal antibody (clone 1B8‐F11; VRMD Inc.) diluted at 1:1000 in PBS containing 1% BSA. After the removal of the antibody, the cells were washed extensively with PBS and incubated with the secondary antibody Alexa 488‐conjugated goat anti‐mouse IgG (A11029, Life Technologies), diluted at 1:500, 1 h at room temperature. Cells were observed using inverted fluorescence microscopy (Zeiss‐Axiovert‐S100, Zeiss), and images were acquired with a digital camera (Zeiss‐Axiocam‐MRC, Zeiss).

A flow cytometric analysis of the transfected cells was also performed with a FACS Canto II (BD Biosciences). Briefly, HEK transfected cells were detached with trypsin 24 h after the transfection, washed, resuspended in cold PBS and counted. One million cells were then fixed, permeabilized and stained as described for the immunofluorescence staining protocol. The percentage of positive cells was determined by recording 50 000 events per sample using a gated strategy for Alexa‐Fluor 488 signals based on the background signal from the cells probed only with the secondary antibody. Data acquisition and analysis were carried out with Diva 9.02 software (BD Bioscience).

### 4.9 Animal Studies and Vaccination Protocol

Female BALB/c mice were bred and maintained at the Molecular Biotechnology Center ‘Guido Tarone’ at the University of Torino. All procedures involving animals complied with European Directive 2010/63 and were approved by the University of Torino's Animal Care and Use Committee and the Italian Ministry of Health. Mice were housed under a 12‐h light/dark cycle with unrestricted access to food and water. The sample size was determined using G*Power software version 3.1.9.7, based on an expected effect size of 0.8, *α* = 0.05 and power = 0.95. A total of 23 female BALB/c mice (8 weeks old) were randomly divided into three groups: 6 mice received the control vaccine (BoHV‐4‐A‐A29), 9 mice received the experimental gDsp‐gFgD and 7 mice the gDsp‐gFgDΔTM vaccine. Each vaccinated mouse received two intraperitoneal injections (200 μL of DMEM containing 10^6^ TCID_50_ of the corresponding viral vector), first at timepoint and again after 14 days. No specific inclusion or exclusion criteria were used, and all animals and data were retained for analysis. Individual mice were treated as experimental units. Two weeks after the second vaccination, mice were anaesthetised by intramuscular injection of Zoletil and RompunT, and blood was collected via cardiac puncture with a 25‐gauge needle, followed by euthanasia. Mice were euthanized by asphyxiation using CO_2_, followed by cervical dislocation. Briefly, groups of mice were transferred in a non‐precharged induction chamber, and CO_2_ was dispensed from a CO_2_ gas distribution system equipped with a pressure regulator, controlling gas flow at about 50% of the chamber volume per minute, to comply with 2020 AVMA Guidelines. CO_2_ flow was maintained for more than 1 min following respiratory arrest, then cervical dislocation was performed to assure euthanasia.

Spleens were harvested and mechanically dissociated using the GentleMACS Octo Dissociator (Miltenyi Biotec), and the resulting cell suspensions were passed through a 70 μm cell strainer. Cells were then centrifuged at 1400 rpm for 10 min, and red blood cells were lysed by incubation in erythrocyte lysis buffer (155 mM NH_4_Cl, 15.8 mM Na_2_CO_3_, 1 mM EDTA, pH 7.3) for 45 s at room temperature. Splenocytes were subsequently washed with PBS and either used immediately for assays or cryopreserved in FBS containing 10% DMSO (Sigma‐Aldrich) at −80°C. Experimenters were not blind to group assignment and outcome assessment.

### 4.10 FACS Analysis

Heparinized blood cells (1 × 10^6^) were blocked with anti‐mouse CD16/CD32 (ThermoFisher Scientific) for 10 min at room temperature to prevent non‐specific Fc binding. Cells were then stained for 30 min at 4°C using the following antibodies: CD45‐VioGreen, CD3‐FITC, CD8‐VioBlue, CD4‐APC‐Vio770, CD49b‐PE (Miltenyi Biotec); and CD44‐PE, CD25‐APC, CD69‐PE/Cy7, CD62L‐APC (BioLegend). 7‐AAD (BD Bioscience) was added, and samples were acquired using a BD FACSVerse (BD Bioscience). Analysis was performed with FlowJO v10.5.3. Briefly, lymphocytes were first gated based on their physical parameters (FSC‐A vs. SSC‐A gating). Doublets were excluded using FSC‐A versus FSC‐W gating, followed by SSC‐A versus SSC‐W gating, and 7‐AAD^+^ dead cells were excluded. CD45^+^ cells were then selected and subdivided using lineage markers: NK cells were identified as CD49b^+^ CD3^−^, while T lymphocytes were defined as CD3^+^ CD49b^−^ and further subdivided into CD4^+^ and CD8^+^ populations. Finally, CD69^+^ and CD25^+^ cells were analysed within the NK, CD4^+^ and CD8^+^ T cell gates to identify activated cells. Moreover, CD44 and CD62L expression on CD4^+^ and CD8^+^‐gated T cells was assessed to identify central memory CD44^+^ CD62L^+^ and effector/effector memory CD44^+^ CD62L^−^ cells. To analyse gD and gF‐specific cellular responses, splenocytes (2 × 10^6^/mL) were cultured for 48 h at 37°C in a 5% CO_2_ incubator with supernatants containing either gDΔTM, gFΔTM‐HA or GFP (negative control). BD GolgiPlug Protein Transport Inhibitor (BD Bioscience, containing Brefeldin A) was added in the last 6 h of culture (1:1000 dilution), then cells were harvested, washed in PBS 0.1% BSA and fixed and permeabilized with the BD Cytofix/Cytoperm kit (BD Bioscience) according to the manufacturer's instructions. Cells were then stained for 30 min at 4°C using LIVE/DEAD Fixable Red Dead Cell Stain Kit (ThermoFisher Scientific), washed and stained for 30 min at 4°C with the following antibodies: CD45‐VioGreen, CD8‐VioBlue, CD4‐APC‐Vio770 (Miltenyi Biotec), CD69‐PE/Cy7 (BioLegend), IFN‐γ‐APC, TNF‐α‐PE and GrzB‐FITC (BD Bioscience). Samples were acquired using a BD FACSVerse. Analysis was performed with FlowJo v10.5.3. Briefly, lymphocytes were first gated based on their physical parameters (FSC‐A vs. SSC‐A). Doublets were excluded using FSC‐A versus FSC‐W, followed by SSC‐A versus SSC‐W gating, and LIVE/DEAD^+^ dead cells were excluded. CD45^+^CD4^+^ and CD45^+^CD8^+^ T‐cell populations were then identified. Finally, CD69^+^ and GrzB^+^ cells, as well as IFN‐γ^+^ and/or TNF‐α^+^ cells, were analysed within the CD4^+^ and CD8^+^ T‐cell gates.

### 4.11 ELISpot Assay

To assess vaccine‐induced T‐cell responses, an ELISpot assay was performed using splenocytes isolated from immunised mice. A total of 5 × 10^5^ splenocytes per well were seeded into 96‐well PVDF membrane plates (ELISpot PLUS: Mouse IFN‐γ (HRP), Mabtech, Cat. No. 3321‐4HPW‐10), pre‐coated with anti‐mouse IFN‐γ capture antibody. Cells were stimulated for 48 h at 37°C in a 5% CO_2_ incubator with supernatants containing either gDΔTM, gFΔTM‐HA GFP (negative control), or with 2 μg/mL concanavalin A (positive control). After incubation, plates were washed and incubated with a biotinylated anti‐IFN‐γ detection antibody (Mabtech, Cat. No. 3321‐4HPW‐10), followed by streptavidin‐HRP (Mabtech). Spots were developed using 3,3′,5,5′‐tetramethylbenzidine (TMB, Merck) substrate solution, and the reaction was stopped by rinsing with deionised water. Spot‐forming cells (SFCs) were quantified using a CTL ImmunoSpot analyser and expressed as the number of SFCs per 10^6^ splenocytes, after subtraction of background levels from unstimulated control wells.

### 4.12 Cytotoxicity Assay

3T3/kB cells were transfected with plasmids encoding for gD, gF or GFP (pgD‐WPRE, pgF and pEGFP‐C1) using Lipofectamine 3000, following the manufacturer's protocol. Twenty‐four hours later, transfected cells were labelled with 2 μm CFSE (ThermoFisher Scientific) for 20 min at 37°C, washed with RPMI‐1640 + 10% FBS and plated (1 × 10^4^ cells/well) in 96‐well plates. Splenocytes from immunised mice were added at a 200:1 ratio, and cells were co‐cultured for 48 h. After incubation, cells were detached and stained with 1 μg/mL 7‐AAD (BD Biosciences) to assess viability. Flow cytometry was conducted using a BD FACSVerse, and data were analysed with FlowJO v10.5.3. Cells first gated based on their physical parameters (FSC‐A vs. SSC‐A gating). Doublets were excluded using FSC‐A versus FSC‐W gating, followed by SSC‐A versus SSC‐W gating. CFSE^+^ target cells were then gated, and the percentage of 7‐AAD^+^ cells was used to determine cell death. Spontaneous death was obtained by culturing target cells without T cells, and maximal cell death was obtained after treatment with 1% saponin. The percentage of specific lysis was calculated using the following formula ([dead targets in sample (%)–spontaneously dead targets (%)]/[dead target maximum (%)–spontaneously dead targets (%)]) × 100.

### 4.13 ELISA Evaluation of gD and gF‐Specific Antibodies

Mouse sera were analysed by an investigator blinded to treatment groups to detect IgG antibodies specific to gD and gF proteins. Ninety‐six wells microplates (MICROLON HIGH BINDING) were coated overnight at 4°C with 50 ng/well of Sec‐gF or ‐gD protein supernatant diluted in 0.1 M carbonate/bicarbonate buffer at pH 9.6. After blocking with 1% BSA, mice sera samples at 1:10 dilution were added to the plate and incubated for 90 min at room temperature. Serum samples were diluted in DMEM/F12 without serum, collected from HEK293 grown for 72 h. After three washing steps in PBS, 50 μL of rabbit anti‐mouse IgG‐HRP conjugated (A9044, Sigma‐Aldrick, Merck) diluted 1:15000 was added to each well and the plate was incubated for 1 h at room temperature. Following the final washing step, the reaction was developed with TMB, stopped with 0.2 M H_2_SO_4_ and read at 450 nm (ChemiDoc, BioRad).

### 4.14 Serum Neutralisation Assay

Mice sera were heat inactivated at 56°C for 30 min. Ten microliters of each serum sample were added to the first row of 96‐well plates. Twenty‐five microliters of cMEM (without FBS) were added to each well, except for the first row, where 40 μL of cMEM were added. Positive and negative virus controls were also included. Serial two‐fold dilutions of each serum from 1:5 to 1:640 were mixed with 25 μL of virus suspension containing 100 TCID_50_ of BoHV‐1 or BRSV. The plates were incubated for 90 min at room temperature and then, 7.5 × 10^4^ AUBEK cells in a volume of 50 μL of cMEM + 10% FBS were added to each well and the plates incubated for 3 days at 37°C with 5% CO_2_. Readings were made by microscopy and/or by 0.5% crystal violet staining of the cell monolayer when CPE were complete in the virus control cultures. The titre of each serum was expressed as the reciprocal (log 2) of the final dilution of serum that completely neutralised viral infectivity.

### 4.15 Statistical Analysis

Data distribution was assessed for normality using the Shapiro–Wilk test, which is appropriate for small sample sizes. Since all datasets satisfied the assumption of normality, comparisons between groups were performed using one‐way ANOVA or two‐way ANOVA, as appropriate, followed by Tukey's test for multiple comparisons. All statistical analyses were performed using GraphPad Prism version 12, and results were considered statistically significant when *p* < 0.05.

## Author Contributions

G.D. and L.C. conceptualised the study and designed the experiments. L.C. and G.D. wrote the paper. A.D.L., S.M., C.C., V.F., E.B., V.M., G.D. and L.C. performed experiments. G.D. and L.C. analysed the data, coordinated and directed the study. All authors have read and agreed to the published version of the manuscript.

## Funding

This work was supported by Università degli Studi di Parma; Università degli Studi di Torino.

## Conflicts of Interest

The authors declare no conflicts of interest.

## Supporting information


**Figure S1:** Expression of chimeric peptides in transfected HEK293T cells. Cells were transfected with pINT2‐CMV‐gDsp‐gF‐gD, pINT2‐CMV‐gDsp‐gF‐gDΔTM, or mock plasmid and stained with anti‐gD monoclonal antibody followed by Alexa Fluor 594‐conjugated secondary antibody for immunofluorescence or Alexa Fluor 488‐conjugated secondary antibody for flow cytometry. Nuclei were counterstained with DAPI. Fluorescence microscopy confirmed chimeric peptides expression, and flow cytometric analysis quantified the percentage of positive cells as shown by density plots.

## Data Availability

All data generated and/or analysed during this study are included in this published article and its Supporting Information file.
