# Supplementary material for: Inducing Antigen‐Specific and Functional Immune Responses in Mice Toward Bovine Herpesvirus 1 and Bovine Respiratory Syncytial Virus by Chimeric Peptides Delivered by Bovine Herpesvirus 4‐Based Vector
Source: Immunology. 2026 May 15;179(1):54–71. doi: 10.1111/imm.70148 (PMC13431852; doi:10.1111/imm.70148)
Supplement: Supplementary file 1 — Figure S1: Expression of chimeric peptides in transfected HEK293T cells. Cells were transfected with pINT2‐CMV‐gDsp‐gF‐gD, pINT2‐CMV‐gDsp‐gF‐gDΔTM, or mock plasmid and stained with anti‐gD monoclonal antibody followed by Alexa Fluor 594‐conjugated secondary antibody for immunofluorescence or Alexa Fluor 488‐conjugated secondary antibody for flow cytometry. Nuclei were counterstained with DAPI. Fluorescence microscopy confirmed chimeric peptides expression, and flow cytometric analysis quantified the percentage of positive cells as shown by density plots. [file IMM-179-54-s001.pdf]

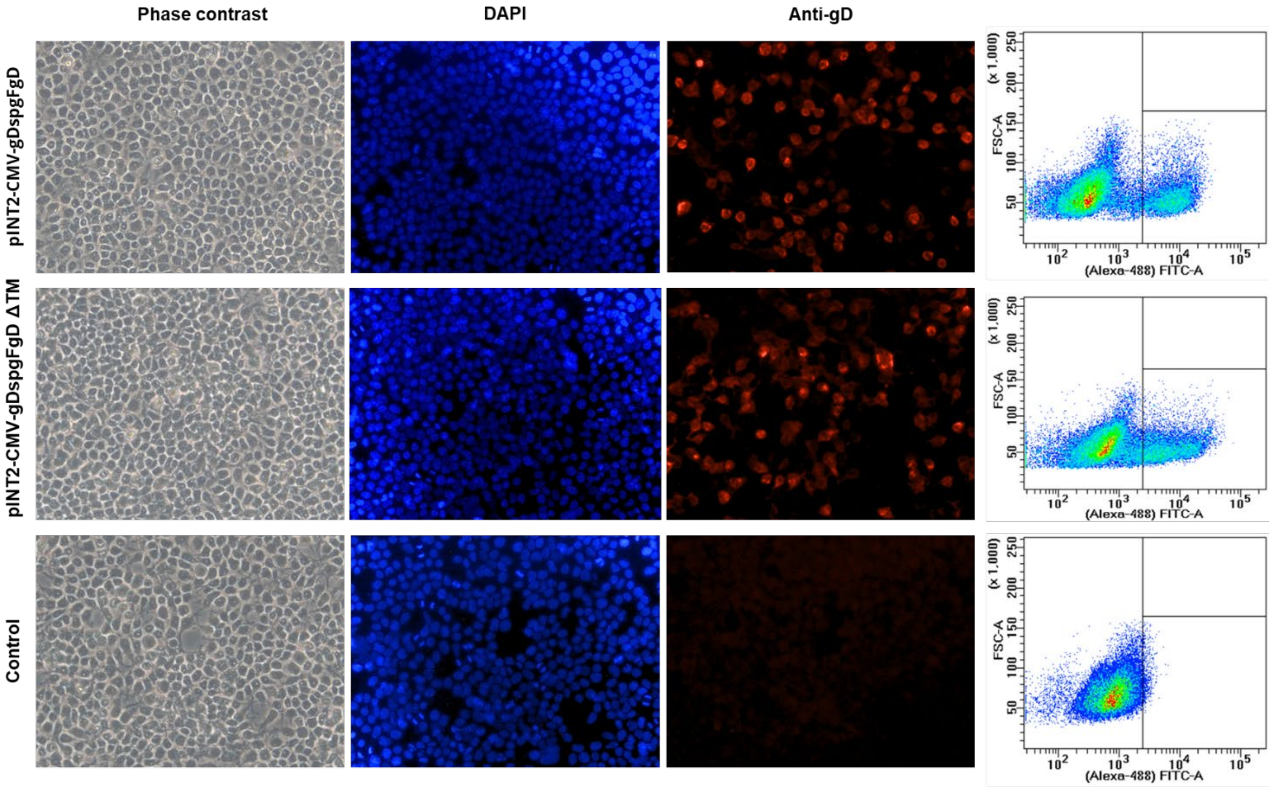

Supplementary Figure 1. Expression of chimeric peptides in transfected HEK293T cells. Cells were transfected with pINT2-CMV-gDsp-gF-gD, pINT2-CMV-gDsp-gF-gDΔTM, or mock plasmid and stained with anti-gD monoclonal antibody followed by Alexa Fluor 594-conjugated secondary antibody for immunofluorescence or Alexa Fluor 488-conjugated secondary antibody for flow cytometry. Nuclei were counterstained with DAPI. Fluorescence microscopy confirmed chimeric peptides expression, and flow cytometric analysis quantified the percentage of positive cells as shown by density plots.
